# Integrated gut microbiota and metabolome signatures revealed by deep metagenomic sequencing in post-stroke cognitive impairment with type 2 diabetes

**DOI:** 10.1128/spectrum.00244-26

**Published:** 2026-05-26

**Authors:** Xiaoye Liu, Lai-Yu Kwok, Wenyi Zhang

**Affiliations:** 1 Key Laboratory of Dairy Biotechnology and Engineering, Ministry of Education, Inner Mongolia Agricultural University 117454 , Hohhot, China; 2 Key Laboratory of Dairy Products Processing, Ministry of Agriculture and Rural Affairs, Inner Mongolia Agricultural University, Hohhot, China; 3 Inner Mongolia Key Laboratory of Dairy Biotechnology and Engineering, Inner Mongolia Agricultural University, Hohhot, China; Huazhong University of Science and Technology, Wuhan, China

**Keywords:** bile acids, ceramides, fungal microbiota, metformin, neuroinflammation, oxidative stress, short-chain fatty acids

## Abstract

Post-stroke cognitive impairment (PSCI) is significantly exacerbated in individuals with type 2 diabetes mellitus (T2DM), yet the underlying gut microbial and metabolic mechanisms remain unclear. In this study, baseline fecal samples from 28 diabetic PSCI (PSCI-DM) patients and 29 matched non-PSCI non-diabetic controls were subjected to deep metagenomic sequencing and untargeted metabolomics. Although alpha diversity was preserved, subtle but meaningful shifts were observed in bacterial and fungal composition. The PSCI-DM group exhibited depletion of beneficial butyrate-producing taxa, including *Lachnospira* spp. and *Butyribacter intestini*, and enrichment of *Butyricimonas virosa*. Five fungal species, including *Torulaspora globosa* and *Pichia kudriavzevii*, were significantly reduced. Metabolomic profiling identified 45 differentially abundant metabolites, with decreases in neuroprotective compounds, such as 9-oxononanoic acid, C16-ceramide, and nootkatone, and increases in metformin and bile acid derivatives. Abundances of microbial functional pathways linked to energy metabolism were elevated, while those involved in cofactor and neurotransmitter precursor synthesis were reduced. Significant correlations were found between specific microbes and metabolites, suggesting coordinated dysregulation across kingdoms. However, only a limited subset of microbial features remained independently associated with cognitive performance. Specifically, metabolites Nb-palmitoyltryptamine and pipecolic acid, and fungal species *Pichia kudriavzevii* showed significant correlations with Montreal cognitive assessment (MoCA) scores for cognitive impairment. These findings reveal a tripartite gut ecosystem signature in PSCI-DM and provide a mechanistic foundation for microbiota-targeted therapeutic strategies.

**IMPORTANCE:**

In the context of type 2 diabetes, post-stroke cognitive impairment represents a clinically prevalent yet mechanistically underexplored condition with limited therapeutic options. This study combined metagenomic sequencing with non-targeted metabolomics to reveal the coordinated dysregulation of bacteria, fungi, and host-related metabolites in the gut of type 2 diabetes mellitus with post-stroke cognitive impairment (PSCI-DM) patients. The research indicates that cognitive impairment is not solely related to the overall decline in microbial diversity, but also involves the targeted reduction of neuroprotective butyrate-producing bacteria, the absence of specific gut fungi, and the corresponding reduction in neural activity and lipid metabolites. These findings collectively establish the gut microbiota-metabolite characteristics of PSCI-DM patients, providing a theoretical basis for targeted probiotic intervention measures to prevent or alleviate cognitive decline in diabetic patients after stroke.

## INTRODUCTION

Type 2 diabetes mellitus (T2DM) is a chronic metabolic disorder characterized by insulin resistance and/or relative insulin deficiency, resulting from a sustained imbalance between carbohydrate intake and metabolic capacity (1). It represents one of the most challenging global health burdens, with clinical hallmarks, including persistent hyperglycemia, impaired insulin secretion, and classic symptoms, such as polyphagia, polydipsia, and polyuria (2). Importantly, T2DM is a major independent risk factor for ischemic stroke. Chronic hyperglycemia promotes atherosclerosis and endothelial dysfunction through multiple interconnected mechanisms, including excessive generation of reactive oxygen species and advanced glycation end products, upregulation of their receptors, and activation of protein kinase C isoenzymes. These pathways collectively impair nitric oxide-mediated vasodilation, amplify pro-inflammatory signaling, and enhance oxidative uptake of low-density lipoprotein in vascular endothelial cells, thereby significantly elevating stroke incidence (3, 4).

A particularly debilitating consequence of stroke is post-stroke cognitive impairment (PSCI), a syndrome defined by the emergence of cognitive deficits within 6 months following a cerebrovascular event. PSCI affects 39% to 47% of stroke survivors (5), triples mortality risk (6), and profoundly diminishes both quality of life and functional independence, imposing substantial emotional and economic burdens on families and healthcare systems (7). Critically, individuals with T2DM exhibit markedly heightened susceptibility to PSCI compared to non-diabetic stroke survivors (8), suggesting that the metabolic dysregulation inherent to diabetes may synergistically exacerbate post-stroke neurocognitive decline.

Beyond traditional vascular mechanisms, growing attention has been directed toward the gut microbiota as a key modulator of brain health via the microbiota-gut-brain axis (9), a bidirectional communication network mediated by neural, endocrine, and immune pathways (10–12). Accumulating evidence links gut microbial dysbiosis to neurodegenerative and cognitive disorders, including Parkinson’s disease (13) and Alzheimer’s disease (14). In the context of metabolic and cerebrovascular disease, distinct microbial alterations have been reported in diabetic cognitive impairment and PSCI when studied as separate conditions. For instance, patients with diabetic microvascular complications frequently exhibit overrepresentation of Proteobacteria and depletion of beneficial short-chain fatty acid (SCFA) producers like *Faecalibacterium* and *Lactobacilli* (15). Similarly, murine models of diabetic cognitive dysfunction show reduced *Rikenellaceae* and elevated *Prevotellaceae* (16).

Independent studies of PSCI also reveal significant gut dysbiosis. Fecal microbiota transplantation from human PSCI patients into post-stroke mice was shown to transfer cognitive deficits, with recipient animals displaying increased *Enterobacteriaceae*, elevated circulating lipopolysaccharide, and reduced butyrate levels, suggesting a causal role for the gut microbiota in cognitive outcomes after stroke (17). A recent cross-sectional study in stroke survivors further confirmed these findings, reporting higher abundances of *Bacteroidaceae* and *Clostridiaceae* in the PSCI patients, while non-PSCI individuals were enriched in *Prevotellaceae* and *Ruminococcaceae* (18). Critically, T2DM appears to amplify post-stroke microbial disruption. Stroke patients with comorbid T2DM show pronounced enrichment of *Enterobacteriaceae*, *Verrucomicrobiota*, and *Klebsiella*, along with elevated levels of the microbiota-derived metabolite phenylacetylglutamine, a compound associated with heightened thrombosis risk (19, 20). Despite these advances, the integrated gut microbial and metabolic signature specifically associated with PSCI in the context of pre-existing T2DM remains uncharacterized.

Given the convergence of metabolic, vascular, and inflammatory insults in this high-risk population, it is plausible that a unique gut ecosystem, encompassing bacteria, fungi, and metabolites, underlies the heightened vulnerability to cognitive decline after stroke. Therefore, this study hypothesized that individuals with T2DM and PSCI harbor a distinct gut microbiota and fecal metabolome profile that distinguish them from controls without diabetes or cognitive impairment, and that these alterations are functionally interrelated and potentially contributory to cognitive dysfunction. To test this hypothesis, a multi-omics approach was employed, integrating deep shotgun metagenomic sequencing and untargeted metabolomics on fecal samples from 28 patients with T2DM and PSCI and 29 age-, sex-, and body mass index-matched participants without diabetes or history of stroke. The aim of this study was to delineate the bacterial, fungal, and metabolic features of the gut ecosystem in diabetic PSCI (PSCI-DM), thereby providing a foundational data set for future mechanistic investigations and the rational design of microbiota-targeted interventions, such as probiotic or metabolite-based therapies.

## MATERIALS AND METHODS

### Subject recruitment and collection of clinical data

Samples were collected from individuals diagnosed with T2DM and PSCI-DM, as well as from non-PSCI-DM control participants, through community-based recruitment. Participants in the PSCI-DM group were required to meet the following inclusion criteria: (i) age between 30 and 80 years, (ii) absence of neurodegenerative diseases, (iii) presence of cognitive dysfunction during the acute phase of stroke, defined as a Montreal cognitive assessment (21) (MoCA) score <25, (iv) a clinical diagnosis of T2DM with a disease duration exceeding 1 year, in accordance with the 2018 American Diabetes Association diagnostic criteria, and (v) capacity to provide informed consent and complete questionnaires and sample collection independently or with assistance from family members.

Exclusion criteria included (i) hemorrhagic stroke or stroke resulting from trauma or surgical intervention, (ii) coexisting neurodegenerative diseases, such as Alzheimer’s disease, (iii) severe cardiovascular, pulmonary, hepatic, or renal diseases or other significant comorbidities that could confound trial outcomes, (iv) inability to complete neuropsychological assessments due to conditions, such as severe aphasia, visual or auditory impairments, or disorders of consciousness affecting written or verbal expression, (v) long-term use of immunosuppressants or recent (within the past month) administration of antibiotics, probiotics, synbiotics, or immunomodulatory agents, and (vi) history of chronic gastrointestinal disorders or malignancies, including peptic ulcers, inflammatory bowel disease, or severe liver disease.

Informed consent was obtained from all participants. Clinical data, including demographic information and MoCA scores, were collected via standardized questionnaires. Age-, sex-, and body mass index-matched control participants without a history of diabetes and stroke were recruited in accordance with the same exclusion criteria. A total of 57 complete data sets, comprising questionnaires and stool samples (28 from the PSCI-DM group and 29 from the non-PSCI-DM group), were obtained with the assistance of participants or their caregivers. Fresh fecal samples were collected using sterile stool samplers, with a DNA preservation solution (Guangdong Longsee Biomedical Co., Ltd., Guangzhou, China) added immediately after collection. Samples were transported on dry ice and stored at −80°C until further processing.

### DNA extraction, metagenomic sequencing, and bioinformatics analysis

Metagenomic DNA was extracted from stool samples using the Magnetic Soil and Stool DNA Kit (DP712; TIANGEN Biotech Co., Ltd., Beijing, China) according to the manufacturer’s protocol. Following quality assessment, DNA was fragmented using a Covaris ultrasonicator. Library preparation was performed through a series of steps: end repair, 3′ adenine tailing, adapter ligation, size selection, PCR amplification, and purification, resulting in final sequencing libraries. Shotgun metagenomic sequencing was achieved on an Illumina Novaseq Xplus platform (Illumina Inc., San Diego, CA, USA).

Raw sequencing data from 57 subjects underwent quality control using Fastp (22) and FastQC (23), yielding a total of 428.06 Gbp of high-quality reads (average: 7.51 Gbp per sample). Clean reads were assembled into contigs using MEGAHIT (24). Contigs longer than 2,000 bp were selected for metagenome-assembled genome (MAG) binning, which was performed in parallel using MetaBAT2 (25), VAMB (26), SemiBin (27), and DAS tools (28) with default parameters. The quality of MAGs was assessed with CheckM (29), and high-quality MAGs were defined as those with ≥80% completeness and ≤5% contamination. Species-level genome bins (SGBs) were then generated using dRep (30) based on strain-level genomic similarity. Taxonomic classification of SGBs was performed by comparison against the Genome Taxonomy Database. Gene abundance was quantified as reads per kilobase per million mapped reads using CoverM (31), and a species abundance table was constructed by aggregating these values across all samples.

Functional annotation was performed using HUMAnN3 (v3.9) (32), with pathway abundance and coverage inferred via alignment to the MetaCyc database. Additionally, fungal composition was analyzed by mapping reads to a custom-built NCBI RefSeq whole-genome database of intestinal fungi using Kraken2 (v2.1.3) (33) and Bracken (v2.9) (34) under default settings, enabling estimation of fungal taxa and their relative sequence abundances.

### Untargeted metabolomics analysis

An accurately weighed portion of each stool sample was transferred into a 2 mL centrifuge tube, and 600 µL of methanol containing an internal standard [2-amino-3-(2-chloro-phenyl)-propionic acid, 4 ppm] was added. The mixture was vortexed for 30 s, followed by the addition of stainless-steel beads and homogenization in a tissue grinder at 50 Hz for 120 s. After ultrasonication at room temperature for 10 min, samples were centrifuged at 12,000 rpm for 10 min. The resulting supernatant was filtered through a 0.22 µm membrane and transferred to autosampler vials for liquid chromatography-mass spectrometry analysis.

Chromatographic separation was performed on a Vanquish UHPLC System (Thermo Fisher Scientific, Inc., Waltham, MA, USA) equipped with an ACQUITY UPLC HSS T3 column (2.1 × 100 mm, 1.8 µm; Waters Corporation, Milford, MA, USA). The column temperature was maintained at 40°C, the flow rate was set to 0.3 mL/min, and the injection volume was 2 µL. In positive ion mode, the mobile phases consisted of (A2) 0.1% formic acid in water and (B2) 0.1% formic acid in acetonitrile; in negative ion mode, (A3) 5 mM ammonium formate and (B3) acetonitrile were used. Gradient elution was applied in both modes. The positive ion gradient elution program is 0–1 min, 10% B2; 1–5 min, 10%–98% B2; 5–6.5 min, 98% B2; 6.5–6.6 min, 98%–10% B2; 6.6–8 min, 10% B2, and the negative ion gradient elution program 0–1 min, 10% B3; 1–5 min, 10%–98% B3; 5–6.5 min, 98% B3; 6.5–6.6 min, 98%–10% B3; 6.6–8 min, 10% B3 (35). Mass spectrometry was conducted on an Orbitrap Exploris 120 mass spectrometer (Thermo Fisher Scientific Inc., Waltham, MA, USA) equipped with an electrospray ionization source, with data acquired in both positive and negative ionization modes.

To ensure data reliability, metabolomics data processing included quality control, normalization, analysis, and visualization. Raw data were converted and peak-aligned using ProteoWizard and the R package XCMS. Metabolite identification was performed by matching mass spectra against multiple databases, including the Human Metabolome Database (36), MassBank (37), LipidMaps (38), mzCloud (39), the Kyoto Encyclopedia of Genes and Genomes database (40), and a laboratory-built library of reference standards.

### Statistical analysis

All statistical analyses and graphical visualizations were performed using R software (v4.5.1) and Adobe Illustrator 2024. Continuous variables were expressed as mean ± standard deviation. Group differences in categorical variables (sex) and continuous variables (age, body mass index) were evaluated using the chi-square test and appropriate non-parametric tests.

Dimensionality reduction was performed using principal component analysis and orthogonal partial least squares discriminant analysis, implemented via the R package Ropls (41). Model overfitting was evaluated using permutation testing. Model performance was assessed by *R*²*Y* (explained variance of the response variable) and *Q*² (predictive ability), with values closer to 1 indicating better model fit and predictive power. Intergroup comparisons of microbial and metabolomic features were conducted using the unpaired Wilcoxon rank-sum test, with *P* values adjusted for multiple testing using the Benjamini-Hochberg procedure. A false discovery rate-adjusted *P* value < 0.05 was considered statistically significant. Correlation networks were constructed and visualized using Cytoscape (v 3.10.1).

## RESULTS

### Demographic data and scale scores

**Fig 1:**
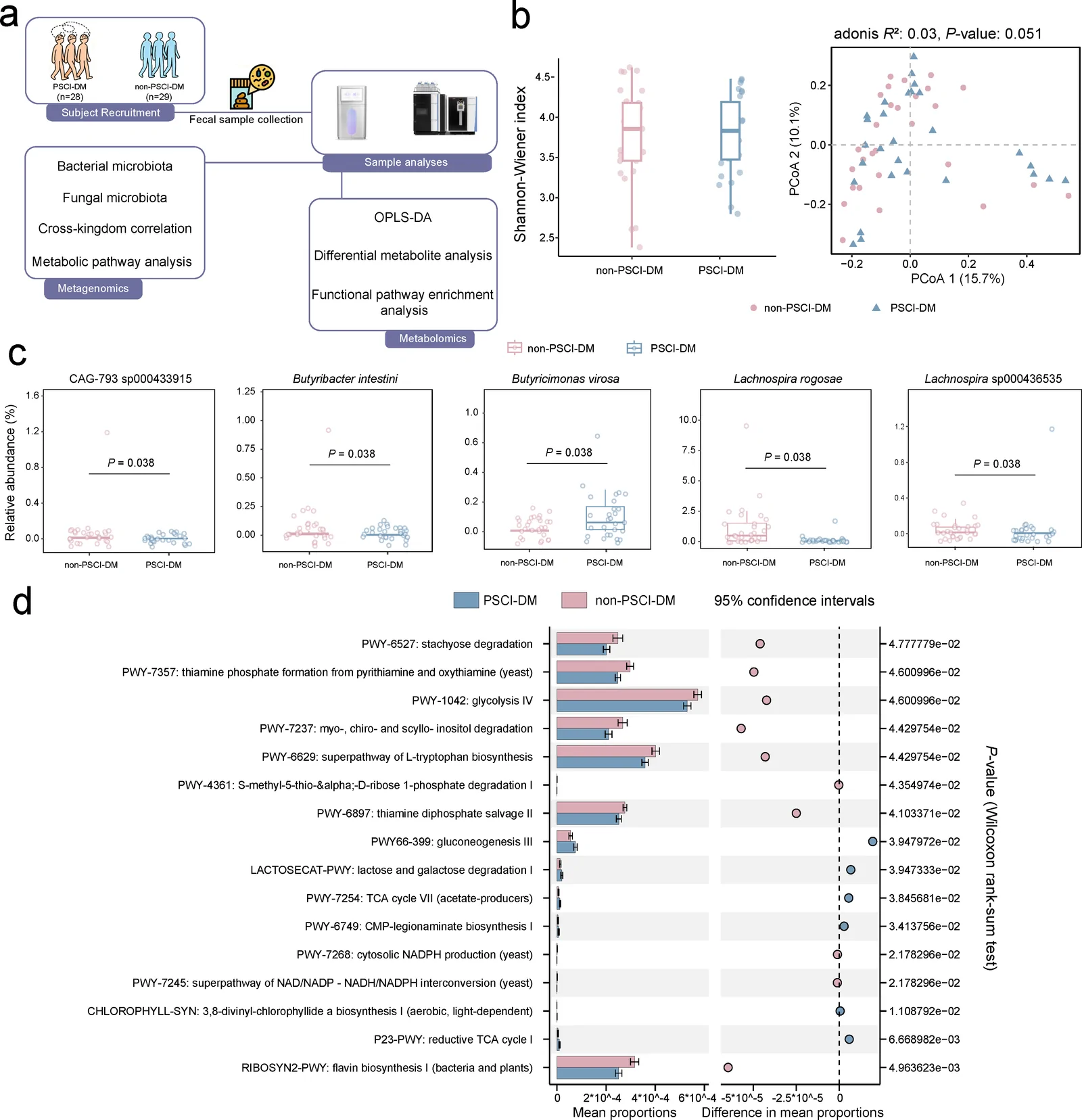
Baseline gut bacterial community features in study participants. (**a**) Study workflow illustrating participant recruitment, sample collection, and multi-omics analyses (fecal metagenomics and metabolomics). (**b**) Alpha diversity (Shannon-Wiener index; left) and beta diversity (principal coordinate analysis, Bray-Curtis distance; PERMANOVA/Adonis test; right) of gut bacterial communities in participants with diabetic post-stroke cognitive impairment (PSCI-DM; *n* = 28) and matched controls without diabetes or stroke (non-PSCI-DM; *n* = 29). (**c**) Relative abundances of five differentially abundant bacterial species. Intergroup differences were evaluated with the Wilcoxon rank-sum test. (**d**) Group-level variation in predicted microbial functions. The left panel shows mean pathway abundance per group, with error bars indicating standard deviation; the right panel displays the difference in means with 95% confidence intervals. Only pathways with *P* < 0.05 are shown. For boxplots, boxes denote interquartile ranges; horizontal lines indicate medians; whiskers extend to 1.5× the interquartile range; outliers are shown as individual points.

The demographic characteristics of the enrolled participants are summarized in Table 1, and the overall study design is shown in Fig. 1a. No significant differences were observed between the PSCI-DM group (*n* = 28) and the non-PSCI-DM control group (*n* = 29) in terms of gender distribution, age, or body mass index. As expected by the study design, a significant difference was detected in MoCA scores between the two groups, consistent with the diagnosis of PSCI in the PSCI-DM cohort.

**TABLE 1:** Demographic and clinical characteristics of participants^*a*^

Characteristic	PSCI-DM (*n* = 28)	Non-PSCI-DM (*n* = 29)	*P* value
Age (years)	68.2 ± 5.80	66.8 ± 6.74	0.4856
Body mass index (kg/m^2^)	26.0 ± 3.20	26.9 ± 2.85	0.2919
Gender (female/male)	14/14	16/13	0.6958
Montreal cognitive assessment score	18.4 ± 3.68	28.4 ± 1.50	<0.01 (7.965e-11)

^*a*^ PSCI-DM, post-stroke cognitive impairment in individuals with type 2 diabetes mellitus; non-PSCI-DM, control participants without stroke, cognitive impairment, or diabetes. Data are presented as mean ± sd. Comparisons between groups were performed using the Wilcoxon rank-sum test for continuous variables and the chi-square test for categorical variables.

### Gut microbiota in PSCI-DM participants

To investigate the gut microbiota profile associated with T2DM and PSCI, fecal metagenomic data from PSCI-DM and non-PSCI-DM participants were compared (Table S1). Alpha diversity, assessed using the Shannon-Wiener index, did not differ significantly between the two groups (*P* > 0.05; Fig. 1b). Beta diversity analysis revealed an almost significant separation in the gut microbial community between groups (*R^2^* = 0.03, *P* = 0.051; Fig. 1b).

Taxonomic profiling at the phylum level identified Bacillota, Bacteroidota, Pseudomonadota, Actinomycetota, Verrucomicrobiota, Desulfobacterota_I, Cyanobacteriota, Methanobacteriota, and Fusobacteriota as the predominant bacterial phyla (Fig. S1). At the species level, 358 SGBs were analyzed, of which five exhibited statistically significant differences in abundance between the groups (*P* < 0.05; Fig. 1c). Compared with the non-PSCI-DM group, the PSCI-DM group showed significantly decreased abundances of *Lachnospira rogosae*, *Butyribacter intestini*, *Lachnospira* sp000436535, and CAG-793 sp000433915. In contrast, *Butyricimonas virosa* was significantly elevated in the PSCI-DM group.

Given that shifts in microbial composition can alter functional metabolic output, pathway-level analyses were performed using the MetaCyc and Kyoto Encyclopedia of Genes and Genomes databases. A total of 504 microbial metabolic pathways were annotated (Table S2), with 16 pathways showing significant intergroup differences (Fig. 1d). Pathways significantly enriched in the non-PSCI-DM group included stachyose degradation, formation of thiamine phosphate from pyrithiamine and oxythiamine (yeast), glycolysis IV, myo-, chiro-, and scyllo-inositol degradation, the superpathway of L-tryptophan biosynthesis, S-methyl-5-thio-alpha, degradation of -d-ribose 1-phosphate I, salvage of thiamine diphosphate II, cytosolic NADPH production (yeast), the superpathway of NAD/NADP–NADH/NADPH interconversion (yeast), and flavin biosynthesis I (bacteria and plants). Conversely, the PSCI-DM group exhibited significantly higher levels of pathways, such as gluconeogenesis III, lactose and galactose degradation I, TCA cycle VII (acetate producers), CMP-lycanate biosynthesis I, 3,8-divinyl-chlorophyllide a biosynthesis I (aerobic, light-dependent), and reductive TCA cycle I.

### Gut mycobiome in PSCI-DM participants

The fungal component of the gut microbiota was also analyzed to assess its potential role in PSCI-DM. A total of 116 fungal species were identified across all samples, spanning three phyla, 14 classes, 23 orders, 34 families, and 66 genera (Table S3; Fig. S2 through S6).

**Fig 2:**
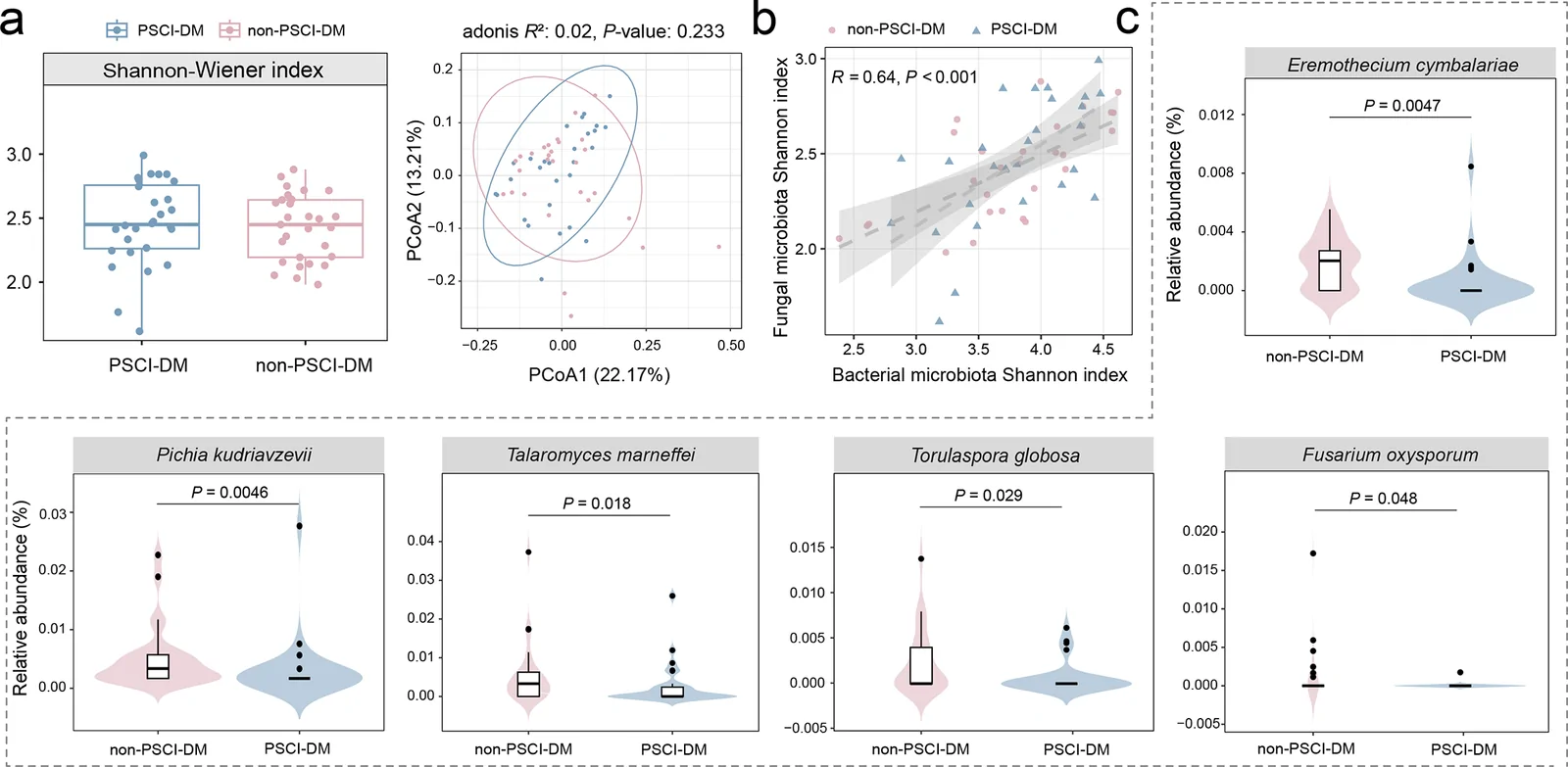
Gut mycobiome structure and cross-kingdom diversity correlations. (**a**) Fungal alpha diversity (Shannon-Wiener diversity index; left) and beta diversity (principal coordinate analysis, Bray-Curtis distance; PERMANOVA/Adonis test; right) between participants with diabetic post-stroke cognitive impairment (PSCI-DM; *n* = 28) and matched controls without diabetes or stroke (non-PSCI-DM; *n* = 29). (**b**) Pearson correlation between bacterial and fungal alpha diversity across all participants. The dashed line represents the line of best fit from linear regression, and the shaded gray band indicates the 95% confidence interval around the regression line. The correlation coefficient and corresponding *P* value are shown. (**c**) Violin plots displaying the distribution density and median of five significantly different fungal species. Intergroup differences were evaluated with the Wilcoxon rank-sum test.

No significant difference in fungal alpha diversity (Shannon-Wiener index) was observed between the PSCI-DM and non-PSCI-DM groups (Fig. 2a). Principal coordinates analysis of beta diversity likewise indicated no statistically significant separation in overall fungal community structure (Fig. 2a). However, a strong positive correlation was detected between bacterial and fungal alpha diversity indices (*R* = 0.64, *P* < 0.001; Fig. 2b), suggesting coordinated ecological dynamics between the two kingdoms.

Further comparison of species-level abundances revealed five fungi that were significantly decreased in the PSCI-DM group: *Eremothecium cymbalariae*, *Pichia kudriavzevii*, *Torulaspora globosa*, *Talaromyces marneffei*, and *Fusarium oxysporum* (Fig. 2c).

### Fecal metabolome in PSCI-DM participants

Fecal metabolomic profiles were analyzed using untargeted liquid chromatography-mass spectrometry in both positive and negative ionization modes. Quality control samples clustered tightly in principal component analysis (Fig. S7), confirming the analytical reproducibility and instrument stability.

**Fig 3:**
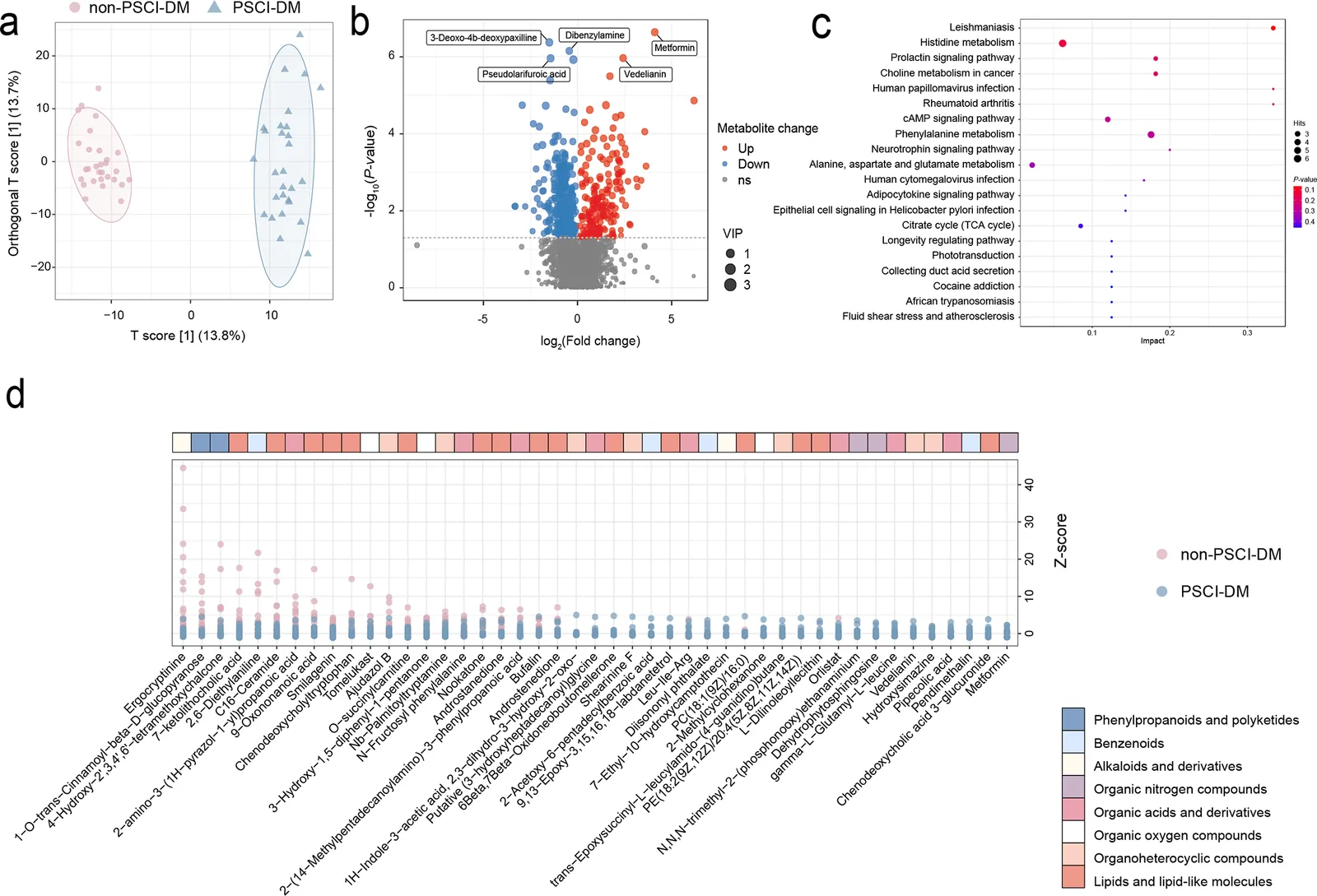
Fecal metabolome distinguishes diabetic post-stroke cognitive impairment from controls. (**a**) Orthogonal partial least squares discriminant analysis score plot showing clear separation between participants with diabetic post-stroke cognitive impairment (PSCI-DM; *n* = 28; triangles) and matched controls without diabetes or stroke (non-PSCI-DM; *n* = 29; circles). (**b**) Volcano plot of differentially abundant metabolites; up (red) = significantly increased; down (blue) = significantly decreased; ns (gray) = no significant change. Metabolites with the highest VIP scores are labeled. (**c**) Top enriched metabolic pathways based on pathway impact and statistical significance, with color intensity representing *P* value and dot size corresponding to the number of metabolites mapped to the pathway. (**d**) Heatmap of Z-scores for significantly altered metabolites between groups, annotated by chemical class (color-coded at the top).

Orthogonal partial least squares discriminant analysis revealed a clear separation between the PSCI-DM and non-PSCI-DM groups (*R*^2^*Y* = 0.978, *Q*^2^ = 0.841; Fig. 3a), indicating robust metabolic differences. Using a variable importance in projection (VIP) threshold >1, 180 metabolites were identified as differentially abundant: 82 were elevated, and 98 were reduced in the PSCI-DM group (Fig. 3b).

Pathway enrichment analysis of these metabolites highlighted involvement in multiple biological processes, including Leishmaniasis, histidine metabolism, prolactin signaling, choline metabolism in cancer, human papillomavirus infection, rheumatoid arthritis, cAMP signaling, phenylalanine metabolism, neurotrophin signaling, alanine/aspartate/glutamate metabolism, human cytomegalovirus infection, adipocytokine signaling, epithelial cell signaling in *Helicobacter pylori* infection, the citrate (TCA) cycle, longevity regulation, phototransduction, collecting duct acid secretion, cocaine addiction, African trypanosomiasis, and fluid shear stress and atherosclerosis (Fig. 3c).

After applying stringent criteria (fold change >2 or <0.5, *P* < 0.05, and false discovery rate correction), 45 metabolites were confirmed as significantly altered (Fig. 3d). These were classified into major chemical categories: phenylpropanoids and polyketides, benzenoids, alkaloids and derivatives, organic nitrogen compounds, organic acids and derivatives, organic oxygen compounds, organoheterocyclic compounds, and lipids and lipid-like molecules. Metabolites significantly increased in the PSCI-DM group included putative (3-hydroxyheptadecanoyl)glycine, Leu-Ile-Arg, PC(18:1(9Z)/16:0), orlistat, pipecolic acid, N,N,N-trimethyl-2-(phosphonooxy)ethanaminium, dehydrophytosphingosine, gamma-L-glutamyl-L-leucine, vedelianin, hydroxysimazine, pendimethalin, chenodeoxycholic acid 3-glucuronide, and metformin. In contrast, metabolites that were significantly reduced in the PSCI-DM group included ergocryptinine, 4-hydroxy-2′,3,4′,6′-tetramethoxychalcone, 7-ketolithocholic acid, 2,6-diethylaniline, C16-ceramide, 9-oxononanoic acid, smilagenin, Nb-palmitoyltryptamine, chenodeoxycholyltryptophan, ajudazol B, O-succinylcarnitine, 3-hydroxy-1,5-diphenyl-1-pentanone, and nootkatone (*P* < 0.05 in all cases).

### Correlation analysis linking gut bacteriome, mycobiome, and metabolome

**Fig 4:**
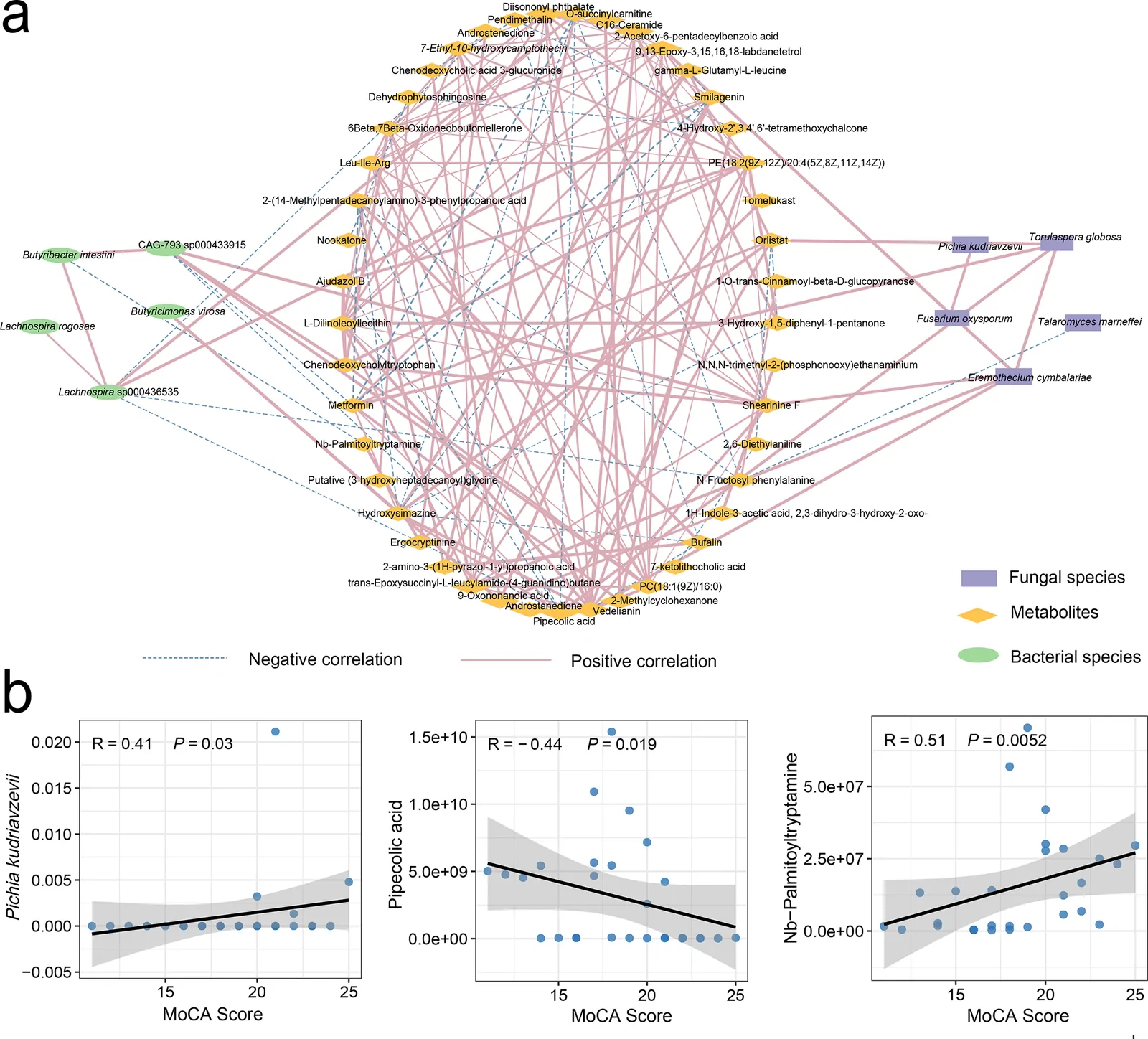
Correlation network in diabetic post-stroke cognitive impairment. (**a**) Spearman correlation network linking bacterial species, fungal species, and fecal metabolites (|*r|* > 0.4, *P* < 0.05). Red solid lines: positive correlations; blue dotted lines: negative correlations. (**b**) Scatter plots showing correlations between MoCA scores and the three features independently associated with cognition, including fungal species *Pichia kudriavzevii*, and the metabolites pipecolic acid and Nb-palmitoyltryptamine. The gray solid lines indicate linear regression fits, with shaded areas representing 95% confidence intervals. The blue dots represent the values on the X and Y axes, respectively.

To explore potential interactions among gut microbes and host metabolism, a correlation network was constructed integrating bacterial SGBs, fungal species, and differential metabolites (Tables S4 and S5; Fig. 4a). Several statistically significant associations (|r| > 0.4, *P* < 0.05) were identified. Notably, 9-oxononanoic acid was positively correlated with *Butyricimonas virosa*. Metformin showed a negative correlation with CAG-793 sp000433915 and a positive correlation with *Torulaspora globosa*. Additionally, *Lachnospira* sp000436535 was positively associated with O-succinylcarnitine. These findings suggest that coordinated alterations in bacterial-fungal-metabolite interactions may contribute to the metabolic dysregulation observed in individuals with PSCI-DM.

To further characterize and understand the multi-omics features related to cognitive performance, we integrated the microbial taxa, functional pathways, and fecal metabolites that were significantly associated with the MoCA score (Fig. 4b). The results revealed that a fungal species, *Pichia kudriavzevii,* and two metabolites, Nb-palmitoyltryptamine and pipecolic acid, showed independent correlations with the cognitive score. Nb-palmitoyltryptamine and *Pichia kudriavzevii* were positively correlated with MoCA (|r| > 0.4, *P* < 0.05), while pipecolic acid was negatively correlated with MoCA. The related network analysis further revealed the coordinated relationships among these cognitive-related features, indicating that the cognitive impairment in the diabetic stroke population is associated with the interrelated microbial and metabolic features.

## DISCUSSION

This integrated metagenomic and metabolomic study reveals a distinct gut microbial and metabolic signature associated with PSCI-DM. While well-matched demographic profiles between groups, PSCI-DM was characterized by coordinated alterations in bacterial and fungal taxa, microbial functional pathways, and fecal metabolites, collectively indicating a tripartite dysbiosis that may contribute to cognitive decline through disruption of the gut-brain axis.

Although alpha diversity was preserved in both bacterial and fungal communities, a marginal separation in bacterial beta diversity (PERMANOVA: *P* = 0.051) and identification of different species suggest a subtle but meaningful community-wide restructuring.

At the species level, five bacterial SGBs were differentially abundant. Notably, *Lachnospira* sp000436535, *Lachnospira rogosae*, and *Butyribacter intestini*, all butyrate-producing members of the Lachnospiraceae family, were significantly depleted. Given the neuroprotective, anti-inflammatory, and blood-brain barrier-stabilizing roles of SCFAs like butyrate, their loss may impair immune homeostasis and exacerbate neuroinflammation (42, 43), which is particularly relevant in the context of diabetes and stroke, both of which are associated with chronic low-grade inflammation (44, 45). Moreover, such a mechanism is conserved across Alzheimer’s disease and mild cognitive impairment (46). In contrast, *Butyricimonas virosa* was significantly elevated in PSCI-DM. Although this species produces butyrate and may enhance the hypoglycemic effects of metformin under certain dietary conditions (47, 48), its expansion in this cohort was not correlated with fecal metformin levels, despite higher metformin detection in current PSCI-DM participants and previous high-fat diet models (49). This suggests that disease-related factors, rather than medication alone, may drive its enrichment. Notably, *Butyricimonas virosa* has also been implicated in bacteremia under conditions of gut barrier disruption (47), raising the possibility of a maladaptive response rather than a beneficial one in PSCI-DM.

The mycobiome, often overlooked in neurological research, was also altered. Five fungal species, including *Pichia kudriavzevii*, *Torulaspora globosa*, and *Fusarium oxysporum*, were significantly reduced. Though their precise roles remain unclear, their depletion may reflect broader ecological instability. The strong positive correlation between bacterial and fungal alpha diversity (*R* = 0.64, *P* < 0.001) further supports interdependent cross-kingdom dynamics (50), implying that disruption in one kingdom may destabilize the other.

Metabolomic profiling identified 45 significantly altered metabolites in the PSCI-DM group, with profound implications for neuroinflammation, oxidative stress, and energy metabolism. Among the most notable reductions was 9-oxononanoic acid, a lipid peroxidation product generated during fatty acid oxidation. Given the well-established role of lipid peroxidation in oxidative stress and mitochondrial dysfunction following cerebral ischemia (51, 52), the decreased levels of this metabolite may reflect either impaired fatty acid metabolism or a blunted oxidative response, both of which could exacerbate neuronal injury and contribute to cognitive decline (53, 54). Similarly, C16-ceramide was reduced in PSCI-DM. In animal models, this long-chain ceramide enhances spatial cognition by activating CaMKII/CREB and Erk signaling pathways (55). Given that these pathways are key downstream effectors of neurotrophin and adipocytokine signaling, its depletion may impair neuroprotective and metabolic regulatory functions in the brain. Additionally, nootkatone, a naturally occurring sesquiterpenoid with demonstrated anti-inflammatory and antioxidant properties, was also markedly reduced. In lipopolysaccharide-stimulated microglial models, nootkatone suppresses pro-inflammatory mediators, such as inducible nitric oxide synthase and cyclooxygenase-2, while enhancing antioxidant enzymes, including NAD(P)H quinone dehydrogenase 1 and heme oxygenase-1 (56). The depletion of nootkatone in PSCI-DM may thus contribute to heightened neuroinflammation (57) and diminished resilience to oxidative stress (58), two core pathological drivers of PSCI-DM. Conversely, bile acid derivatives, such as chenodeoxycholic acid 3-glucuronide, were increased, while 7-ketolithocholic acid decreased, suggesting dysregulated bile acid signaling through farnesoid X receptor and Takeda G-protein coupled receptor 5, which modulate neuroprotection and microglial activation (59).

Functional pathway analysis further revealed a microbial metabolic shift toward energy generation (e.g., gluconeogenesis and the reductive tricarboxylic acid cycle) and away from biosynthetic pathways for essential cofactors (e.g., thiamine, flavins) and tryptophan, a precursor for neuromodulatory serotonin and kynurenine metabolites. This may compromise neurotransmitter balance and redox homeostasis in the brain, creating a metabolic environment conducive to cognitive decline.

A major strength of this study lies in its multi-omics integration, capturing bacteria, fungi, and metabolites at high taxonomic resolution using MAGs and SGBs. Although inter-group comparisons revealed extensive differences in the composition of microorganisms and metabolites between PSCI-DM and non-PSCI-DM, most differential species and metabolites were not directly associated with cognitive variations. Instead, two metabolites (pipecolic acid and Nb-palmitoyltryptamine) and a fungal species (*Pichia kudriavzevii*) were significantly correlated with the MoCA score, emphasizing that the cognitive impairment in stroke with diabetes may be related to restricted and specific microbial-metabolic characteristics rather than widespread dysbiosis of the microbiota. Nevertheless, limitations include the cross-sectional design, which precludes causal inference; unmeasured confounders, such as diet, blood glucose, blood pressure, lipid profiles, and detailed medication history; and a modest sample size that may limit generalizability. Future validation in longitudinal cohorts and functional studies in gnotobiotic models is warranted. Importantly, the current design does not allow the independent effects of diabetes, stroke, and post-stroke cognitive impairment to be fully distinguished, and potential confounding factors may exist. Nevertheless, through integrated multi-omics analysis, this study provides valuable insights into the overall gut microbiota and metabolome signatures in patients with post-stroke cognitive impairment and type 2 diabetes, generating hypotheses and laying the groundwork for future larger, stratified studies.

In conclusion, PSCI-DM is not defined by simple diversity loss but by a rewiring of gut microbial and metabolic networks. The depletion of beneficial taxa, mycobiome alterations, and shifts in neuroactive metabolites collectively create a permissive environment for cognitive decline. These findings provide a foundation for microbiota-targeted interventions, such as precision probiotics, prebiotics, or metabolite supplementation, to mitigate PSCI in high-risk diabetic populations.

## Data Availability

The metagenomics data have been uploaded to the National Genomics Data Center GSA data repository under the BioProject ID number PRJCA051818.

## SUPPLEMENTAL MATERIAL

Supplemental figures spectrum.00244-26-s0001.docx Figures S1 to S7.

Supplemental tables spectrum.00244-26-s0002.xlsx Tables S1 to S5.

ASM does not own the copyrights to Supplemental Material that may be linked to, or accessed through, an article. The authors have granted ASM a non-exclusive, world-wide license to publish the Supplemental Material files. Please contact the corresponding author directly for reuse.
