# Supplementary material for: Integrated gut microbiota and metabolome signatures revealed by deep metagenomic sequencing in post-stroke cognitive impairment with type 2 diabetes
Source: Microbiol Spectr. 2026 May 26;14(7):e00244-26. doi: 10.1128/spectrum.00244-26 (PMC13340151; doi:10.1128/spectrum.00244-26)
Supplement: Supplemental figures — Figures S1 to S7. [file spectrum.00244-26-s0001.docx]

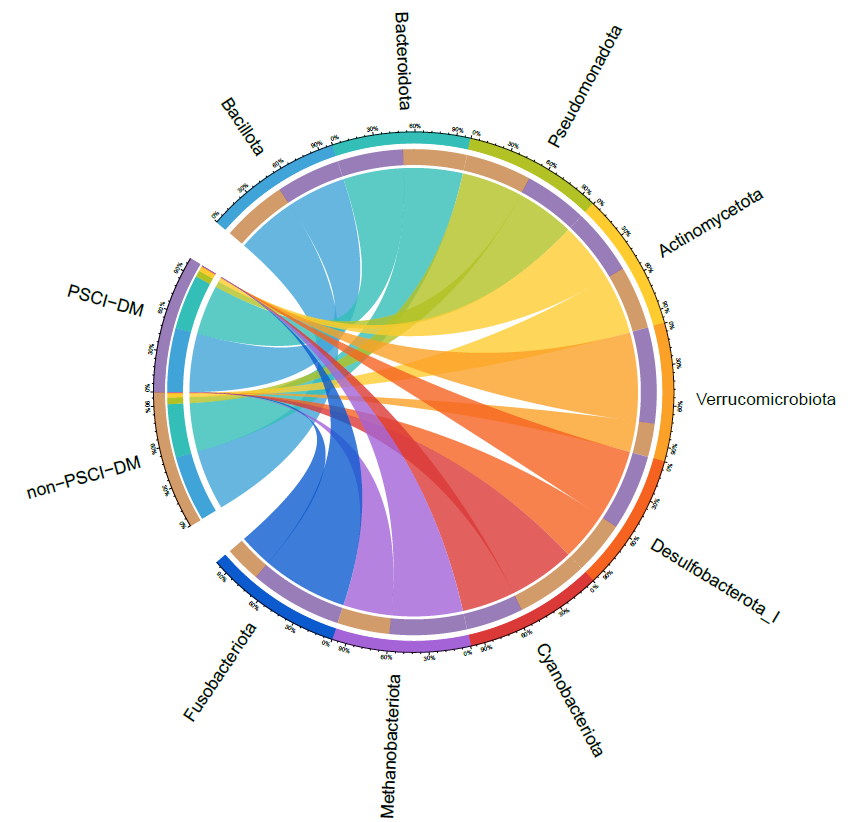


**Fig. S1 Phylum-level gut microbiota composition.** The Circos plot displays the relative abundance of dominant phyla (such as Bacteroidetes, Bacillota) in the Type 2 diabetes mellitus with post-stroke cognitive impairment (PSCI-DM) and non-PSCI-DM.


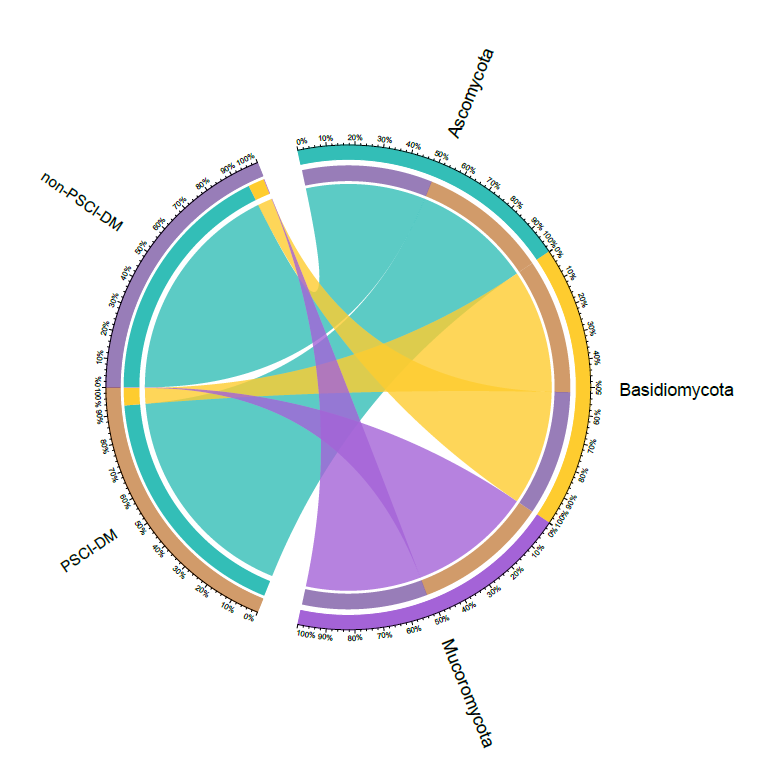


**Fig. S2 Phylum-level gut fungal microbiota composition.** The Circos plot displays the relative abundance of fungal dominant phyla (include Ascomycota, Basidiomycota, Mucoromycota) in the PSCI-DM and non-PSCI-DM.


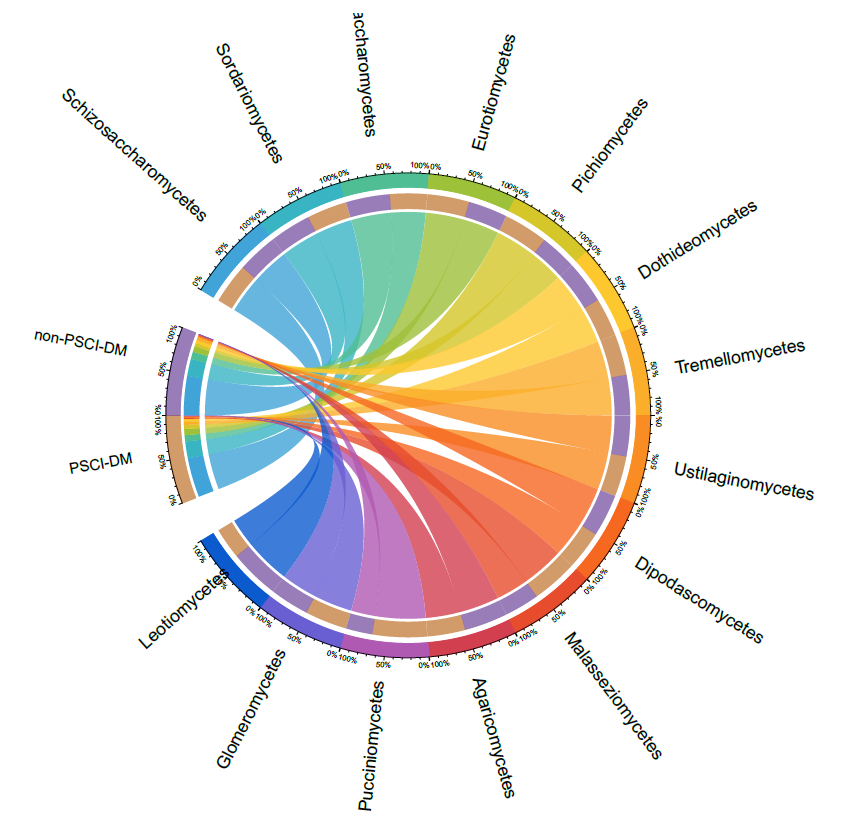


**Fig. S3 Class-level gut fungal microbiota composition.** The Circos plot displays the relative abundance of fungal dominant class in the PSCI-DM and non-PSCI-DM.


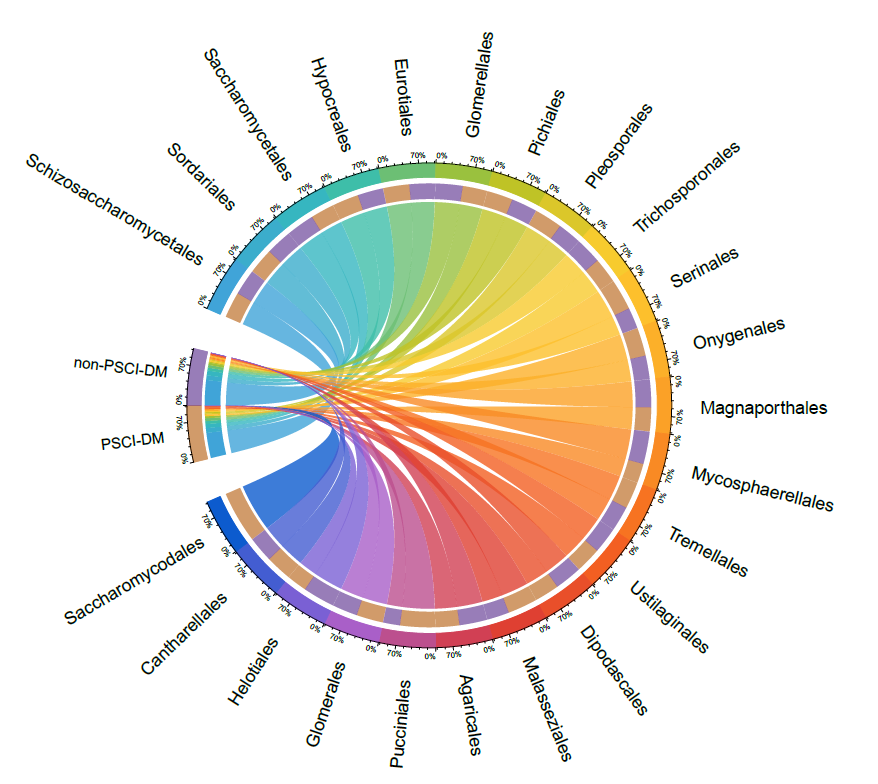


**Fig. S4 Order-level gut fungal microbiota composition.** The Circos plot displays the relative abundance of fungal dominant order in the PSCI-DM and non-PSCI-DM.


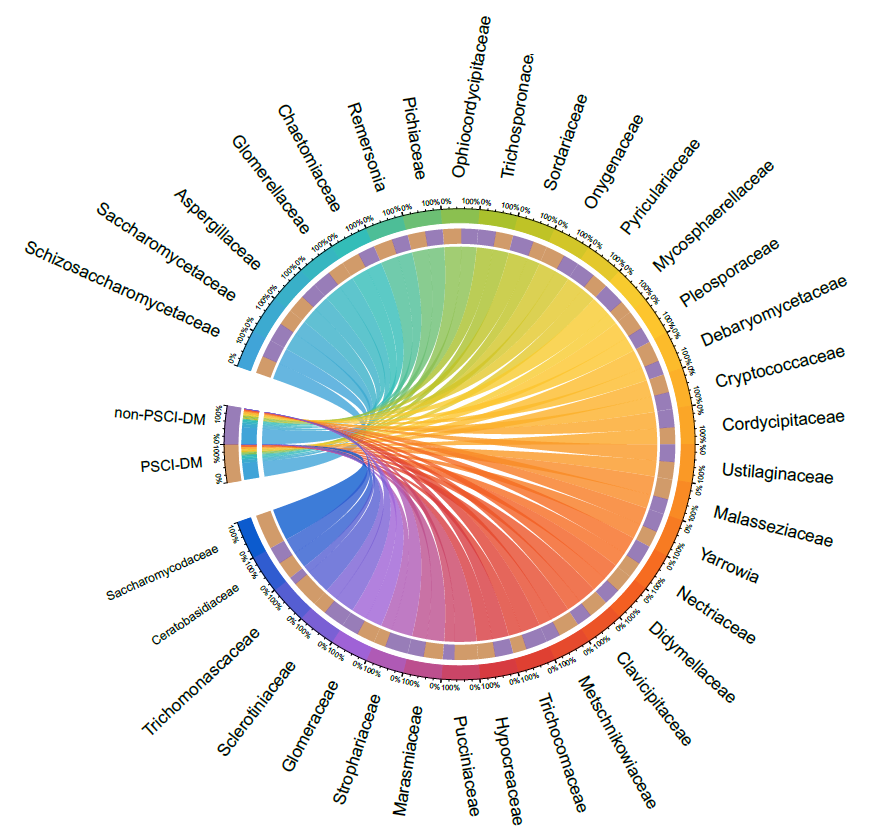


**Fig. S5 Family-level gut fungal microbiota composition.** The Circos plot displays the relative abundance of fungal dominant family in the PSCI-DM and non-PSCI-DM.


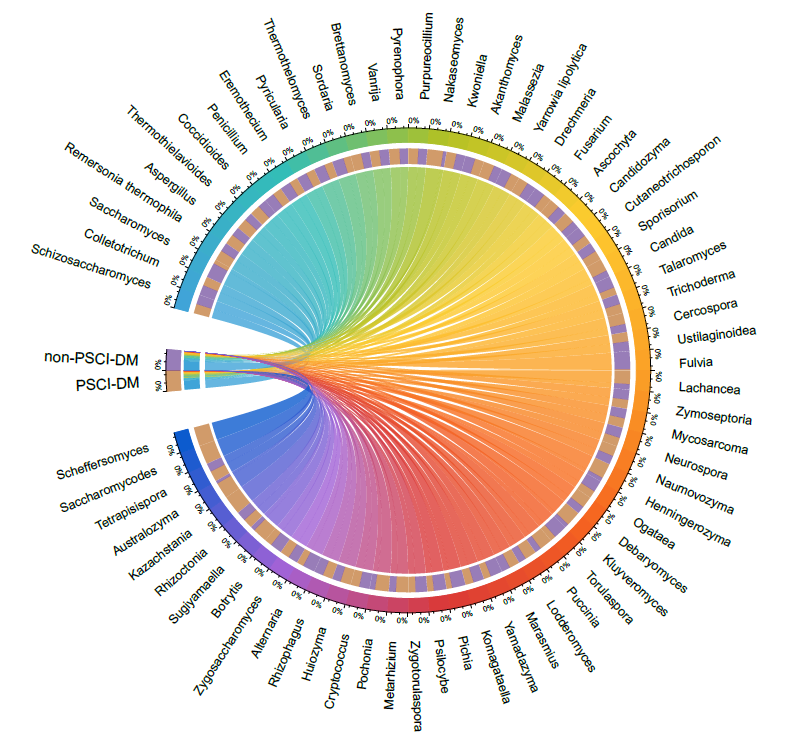


**Fig. S6 Genus-level gut fungal microbiota composition.** The Circos plot displays the relative abundance of fungal dominant genus in the PSCI-DM and non-PSCI-DM.


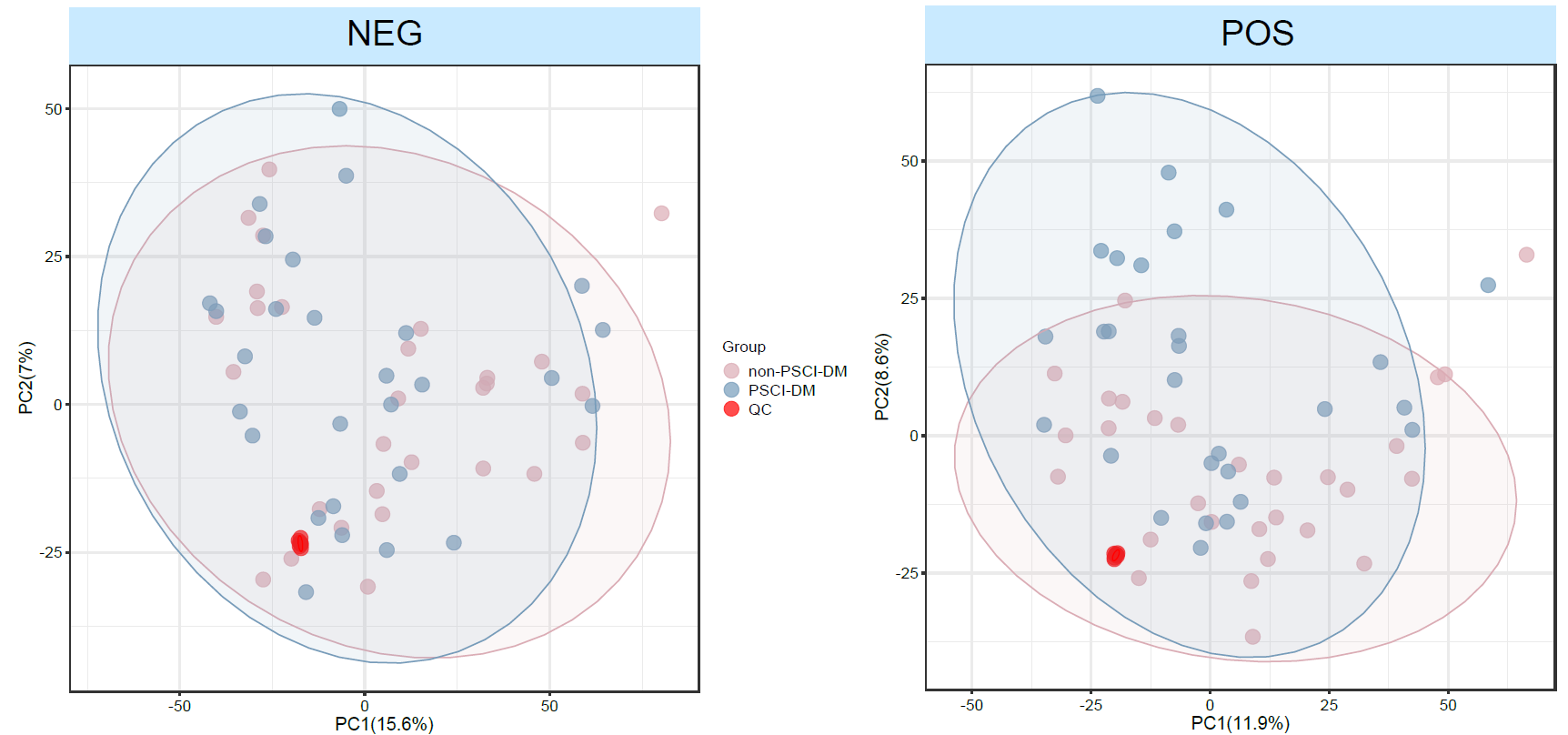


**Fig. S7 Principal Component Analysis (PCA) of fecal metabolomes with quality control (QC) validation in negative ions and positive ions.** The PCA score plot shows clustering of QC samples and PSCI-DM and non-PSCI-DM.
